# Comparative analysis of HIV-1-based lentiviral vectors bearing lyssavirus glycoproteins for neuronal gene transfer

**DOI:** 10.1186/1479-0556-7-1

**Published:** 2009-01-13

**Authors:** Thais Federici, Robert Kutner, Xian-Yang Zhang, Hitoshi Kuroda, Noël Tordo, Nicholas M Boulis, Jakob Reiser

**Affiliations:** 1 Department of Neurosurgery, Emory University, Atlanta, GA, USA; 2 Gene Therapy Program, Louisiana State University Health Sciences Center, New Orleans, LA, USA; 3 Unit Antiviral Strategies, CNRS-URA 3015, Institut Pasteur, Paris, France; 4 U.S. Food and Drug Administration, Center for Biologics Evaluation and Research, Division of Cellular and Gene Therapies, Bethesda, MD, USA

## Abstract

**Background:**

The delivery of therapeutic genes to the central nervous system (CNS) using viral vectors represents an appealing strategy for the treatment of nerve injury and disorders of the CNS. Important factors determining CNS targeting include tropism of the viral vectors and retrograde transport of the vector particles. Retrograde transport of equine anemia virus (EIAV)-based lentiviral vectors pseudotyped with the glycoprotein derived from the Rabies virus RabERA strain from peripheral muscle to spinal motor neurons (MNs) was previously reported. Despite therapeutic effects achieved in mouse models of amyotrophic lateral sclerosis (ALS) and spinal muscular atrophy (SMA), the efficiency of this approach needs to be improved for clinical translation. To date there has not been a quantitative assessment of pseudotyped HIV-1-based lentiviral vectors to transduce MNs. Here, we describe quantitative tests to analyze the retrograde transport capacity of HIV-1 vectors pseudotyped with the G glycoprotein derived from Rabies and Rabies-related viruses (Lyssaviruses).

**Methods:**

With a view toward optimizing the retrograde transport properties of HIV-1-based lentiviral vectors, we compared the glycoproteins from different enveloped viruses belonging to the *Rhabdoviridae *family, genus *Lyssavirus*, and evaluated their ability to transduce specific cell populations and promote retrograde axonal transport. We first tested the transduction performance of these pseudotypes *in vitro *in SH-SY5Y neuroblastoma cells, NSC-34 neuroblastoma-spinal cord hybrid cells, and primary mixed spinal cord and pure astrocyte cultures. We then analyzed the uptake and retrograde transport of these pseudotyped vectors *in vitro*, using Campenot chambers. Finally, intraneural injections were performed to evaluate the *in vivo *retrograde axonal transport of these pseudotypes.

**Results:**

Both the *in vitro *and *in vivo *studies demonstrated that lentiviral vectors pseudotyped with the glycoprotein derived from the Rabies virus PV strain possessed the best performance and neuronal tropism among the vectors tested.

**Conclusion:**

Our results indicate that HIV-1-based lentiviral vectors pseudotyped with the Rabies PV glycoprotein might provide important vehicles for CNS targeting by peripheral injection in the treatment of motor neuron diseases (MND), pain, and neuropathy.

## Background

Lentiviral and adeno-associated viral (AAV) vectors are the most promising vectors in the field of gene transfer for neurodegenerative diseases [1-3]. The larger cloning capacity of lentiviral vectors, however, makes them more suitable for therapeutic purposes. A variety of primate lentiviral vectors including vectors based on HIV-1 [4,5] and simian immunodeficiency virus (SIV) [6], or on non-primate lentiviral vectors such as EIAV [7,8] and feline immunodeficiency virus (FIV) [9] have been shown to mediate significant transgene delivery in the mammalian nervous system. Finally, lentiviral-based gene transfer strategies have been widely tested for the treatment of neurological disorders, such as Parkinson's disease (PD), Alzheimer's disease (AD), Huntington's disease (HD), spinal cord injury, metabolic disorders, and MND [2,10-12]. We have recently reviewed the existing gene therapy strategies for MND [13]. Although direct vector injection has been reported [8,14], gene delivery to spinal cord MNs by retrograde axonal transport following muscle injection is the main strategy being currently explored [8,15-17].

It has been well documented that the tropism of lentiviral vectors can be altered using pseudotyping strategies, which consist in the replacement of the vector envelope glycoprotein for alternative glycoproteins derived from other enveloped viruses. A wide range of viral envelope glycoproteins, including those from Rabies and Mokola viruses have been used for pseudotyping [18]. However, considerable differences in the brain transduction patterns were demonstrated even between related Lyssavirus-derived glycoproteins. For example, transduction of the mouse striatum by HIV-1 vectors pseudotyped with the glycoprotein derived from a Zimbabwean Mokola virus was inefficient [19,20], while HIV-1 vectors bearing an Ethiopian Mokola virus glycoprotein revealed robust transduction [21,22].

To date there has not been a quantitative assessment of pseudotyped HIV-1-based lentiviral vectors to transduce MNs. In the present study, we compared four different titer-adjusted HIV-1 vector pseudotypes bearing envelope glycoproteins from Rabies-related viruses, such as the European Bat Lyssavirus (EBL1), the Lagos Bat Lyssavirus (LagNGA), the Duvenhage (DuvSAF1) Lyssavirus, and Rabies PV virus [23] in terms of their transduction efficiencies *in vitro *and *in vivo*. We found that lentiviral vectors pseudotyped with the Rabies PV G-glycoprotein had the best performance in all the assays conducted, being, therefore, a promising candidate for future gene delivery strategies involving pseudotyped HIV-1-based lentiviral vectors.

## Methods

### Cell culture

#### Cell lines

293T cells (CRL-11268), human osteosarcoma (HOS) cells (CRL-1543) and BHK21 cells (CCL-10) were obtained from the American Type Culture Collection (ATCC). DMEM/10% FBS (Invitrogen) was used to propagate 293T and HOS cells and Eagle's Minimum Essential Medium (EMEM)/10% FBS for BHK21 cells. Human neuroblastoma SH-SY5Y cells (ATCC CRL-2266) were grown to 70% confluence and maintained in a growth medium containing a 1:1 mixture of EMEM and Ham's F12 medium (90%) (Invitrogen), supplemented with 10% FBS (HyClone). Medium was renewed every 2 days. The NSC-34 neuroblastoma-spinal cord hybrid cell line [24] was provided by Dr. Neil Cashman, Toronto. NSC-34 cells were propagated in DMEM/10% FBS.

#### Astrocyte cultures

Astrocytes were isolated from embryonic day 15 mouse cerebral cortices and kept in 75-cm^2 ^flasks coated with poly-D-lysine containing a 1:1 mixture of DMEM and Ham's F12 medium supplemented with 5% FBS, 5% horse serum, 2 mM GlutaMAX, 100 U/ml penicillin and 100 mg/ml streptomycin. For transduction, cells were harvested and plated into 8-well chamber slides (Nalge Nunc) coated with poly-D-lysine.

#### Mixed spinal cord cultures

Spinal cords were obtained under sterile conditions from 15-day Sprague Dawley rat embryos. Dorsal root ganglia (DRGs) and perineural membranes were removed and cords cut into approximately 2-mm sections, which were then triturated and dissociated in a 0.05% trypsin/0.53 mM EDTA solution. Cells were collected, centrifuged for 5 min at 1800 rpm, and resuspended in complete growth medium made in supplemented Neurobasal Medium (Invitrogen-GIBCO). Cells were plated on glass coverslips in multi-well cell culture plates pre-coated with poly-L-lysine (24 h, 0.005% in H_2_O).

#### Compartmentalized chambers

DRGs were carefully removed from 15-day-old Sprague-Dawley rat embryo cords and cultured following an established protocol [25]. Briefly, DRG explants were plated into the inner compartments of Campenot compartmentalized chambers [26] in a small volume of medium and allowed to adhere for two hours. The chambers were prepared as follows: 35-mm cell culture dishes were pre-coated with a diluted collagen solution (three parts of sterile distilled H_2_O to one part of rat tail collagen type I – 2 mg/ml – Roche Diagnostics Corp.). Scratches were then made in the collagen substratum using a pin rake, to guide axon growth. A drop of medium was placed onto the plate before setting the divider onto the culture dish, in order to facilitate axon growth underneath the silicon grease barriers. Teflon chamber dividers (Tyler Research) were then carefully attached to the collagen-coated dishes using silicone vacuum grease (Dow Corning). After 1 h in the incubator, the inner compartments of the chambers were filled with culture medium to check for leakage. Chambers that showed any leakage were discarded and those that appeared well-sealed were filled with culture medium. Fluorescence was also used to demonstrate the absence of leakage between chambers, by adding a fluorescent tracer (DiI – Molecular Probes, Invitrogen Corporation) in the inner compartment. The compartments were then filled with growth medium, consisting of Neurobasal medium supplemented with B-27 additive (Invitrogen), 50 ng/ml of Nerve Growth Factor 2.5S (NGF) (Invitrogen), and 40 μM of 5'-Fluoro-2'-deoxyuridine – FUDR (Sigma-Aldrich). The outer compartments were filled with growth medium supplemented with 100–200 ng/ml of NGF to coax neurite growth into these compartments. Cells were re-fed every other day. The outer compartments were also re-filled every other day with growth medium supplemented with NGF.

#### β-Gal staining and immunocytochemistry (ICC)

SH-SY5Y cells and mixed spinal cord cell cultures were processed for β-Gal staining 3 days after transduction using an X-Gal staining kit (Invitrogen). In order to identify neurons and glia in the cultures transduced with the EGFP-encoding vectors, ICC was performed with antibody directed against microtubule associated protein 2 (Map-2) and glial fibrillary acidic protein (GFAP). The Map-2 antibody (Covance Research Products) was used at a concentration of 1:10,000 and anti-GFAP antibody (Promega) at a concentration of 1:1000. Fluorescent cells were visualized using a Nikon E400 upright microscope.

#### Lentiviral vectors

#### Plasmid constructs

The pNL-EGFP/CMV/WPREΔ U3 lentivirus vector plasmid was described before [27]. In the pNL-EGFP/lacZco/WPREΔ U3 lentiviral vector plasmid [28], a codon-optimized lacZ gene sequence encoding β-galactosidase (β-Gal) [29] was used to replace the EGFP transgene sequence. The VSV-G-encoding pLTR-G plasmid was described before [30]. Plasmids encoding glycoproteins of the Rabies virus PV strain (GenBank Accession number: A14671), Duvenhage virus (DuvSAF2 strain) (GenBank Accession number: AF298147), European bat Lyssavirus (EBL1FRA strain) (GenBank Accession number: AF298143) and Lagos bat Lyssavirus (LagNGA strain) (GenBank Accession number: AF298148) [23] were constructed as follows: Total RNA was extracted from BHK-21 cells infected with the various Lyssavirus isolates and reverse-transcribed using an ImProm-II Reverse Transcription System (Promega) and primers corresponding to the 3'-untranslated regions of the various viral RNAs. The primers used for reverse transcription were: Rabies PV: 5' CGG GAT CCG GCC AGC TCT CAC AGT CCG GT; DuvSAF: 5' CGG GAT CCC TCT CAC TCC CTT GTT GAT GG; EBL1: 5' CGG GAT CCT GCT TAT GAC TCA CAA GTA GT; LagNGA: 5' GGA ATT CTT GTT ACC ATG AGT CAA CTA AAA. The glycoprotein coding regions were subsequently PCR amplified using AccuPrime™ Pfx DNA Polymerase (Invitrogen) and subcloned into pLTR [30] to yield pLTR-PV, pLTR-DuvSAF, pLTR-EBL1 and pLTR-LagNGA, respectively. The primers used for PCR were as follows: Rabies PV: 5' GGA ATT CCA AGG AAA GAT GGT TCC TCA G (sense), 5' CGG GAT CCG GCC AGC TCT CAC AGT CCG GT (antisense); DuvSAF: 5' GGA ATT CAC CAT GCC ACT CAA TGC AGT CA (sense), 5' CGG GAT CCC TCT CAC TCC CTT GTT GAT GG (antisense); EBL1: 5' GGA ATT CAC CAT GTT ACT CTC TAC CGC CA (sense), 5' CGG GAT CCT GCT TAT GAC TCA CAA GTA GT (antisense); LagNGA: 5' CCC CCG GGA TCA GAC ATT AGA GCT ACC CT (sense), 5' GGA ATT CTT GTT ACC ATG AGT CAA CTA AAA (antisense). All glycoprotein-encoding sequences were analyzed by DNA sequencing.

### Sciatic nerve injections

All animal procedures were approved by the Animal Care and Use Committee and strictly adhered to the requirement set forth by the Guide for the Care and Use of Laboratory Animals. Sciatic nerve injections were performed as previously described [37]. Briefly, adult male Sprague Dawley rats (Harlan), weighing 275–300 g, were anesthetized with nose-cone isoflurane (2% in O_2_). The lateral surface of the right thigh was shaved, sterilized, and a skin incision was made parallel to the femur. Using a dissecting microscope (StereoZoom 6; Leica), the sciatic nerve was exposed and a 5-0 silk suture was tied to provide gentle counter traction during the nerve injection. To crush the nerve, a clamp was placed around the nerve immediately before the tie, closed to the first lock and then released. Using an automatic microinjector (Nanoject II; Drummond), nerves were injected with 2 μl of the different β-Gal-encoding pseudotypes (n = 5/group), adjusted at 1.25 × 10^4 ^TU per μl. After injection, the femoral musculature was reapproximated and the skin stapled. Animals were monitored for limb weakness, paralysis, and infection, as well as for symptoms of neurogenic pain including limb mutilation. Animals were euthanized 4 weeks after injections.

#### β-Gal staining and histology

For histological analyses, rats were deeply anesthetized with sodium pentobarbital (100 mg/kg, I.P.) and transcardially perfused with a 0.9% saline solution, followed by 4% paraformaldehyde (Sigma-Aldrich) in phosphate buffer saline (PBS), pH 7.4. Sciatic nerves, DRGs, and spinal cords were dissected, post-fixed for 24 h, and transferred to a 30% solution of sucrose. Tissues were first processed for β-Gal staining and then frozen in optimal cutting temperature gel (OCT; Sakura Finetek USA) and stored until histological processing. Nerves, DRGs, and spinal cords were cut at 25 μm in transverse sections on a cryostat (Leica Microsystems) and mounted onto slides. Counter-staining was performed on transverse sections using Eosin. Finally, the slides were mounted with coverslips using Permount mounting medium (Fisher Scientific).

### Quantitative analysis

All the experiments were performed at least in triplicate. In order to derive the percentage of transduced cells, images were acquired by a Nikon E400 microscope using a DS-Qi1 High-sensitivity Cooled CCD camera and analyzed using the NIS-Elements imaging software (Nikon Instruments, Inc). Cells were counted in 10 randomly fields per slide and the mean percentages of positive cells were calculated. For quantification of transduction in the chambers, the fluorescence pixel intensity within the DRG cell bodies was quantified. Data is expressed as mean ± standard deviation (SD).

### Statistical Analysis

Individual conditions (such as the effect of different vector treatments) were compared and statistically analyzed for significance with one-way analysis of variance (ANOVA) and *post hoc *Tukey tests using the Jandel SigmaStat software. Combined effects of vector treatment/MOI or vector treatment/astrocyte culture (mixed or pure) were compared with a two-way ANOVA test.

## Results

### Titration of lentiviral vectors bearing lyssavirus glycoproteins

To compare the performance of the various pseudotypes it was important to accurately adjust the vector's multiplicities of infection (MOI) prior to transduction. This brought up the issue as to what technique would be most suitable to determine the titers. Currently, there are different measures available to determine lentiviral vector titers [18]. Some of them rely on the number of vector particles present in a vector stock based on strong-stop cDNA or on viral RNA present in virions [38,39]. Others are based on the amount of virus proteins present in vector cores such as p24 Gag [40]. Functional titration assays are based on vector-encoded reporter gene expression. For example, vectors encoding β-Gal have been titrated using X-Gal staining [28]. Also significant for titration is the cell line used as receptors for a given pseudotype may vary from cell line to cell line, possibly producing a falsely depressed titer.

To accurately compare the performance of the various pseudotypes, we tested two different titration protocols to adjust vector MOIs including protocols based on functional titers and protocols based on particle titers.

#### Transduction efficiencies of titer-adjusted lentiviral vector pseudotypes using functional titers

**Figure 1:**
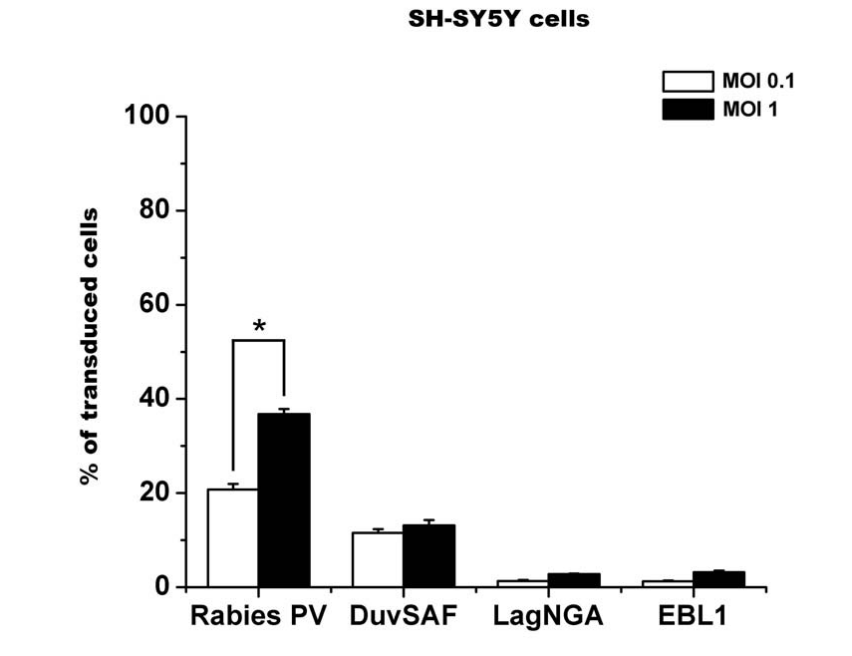
**Transduction of SH-SY5Y cells using lentiviral vectors pseudotyped with different lyssavirus glycoproteins**. SH-SY5Y neuroblastoma cells were transduced using β-Gal-encoding lentiviral vectors pseudotyped with Rabies-PV, DuvSAF, LagNGA, and EBL1 glycoproteins. MOIs were adjusted based on functional titers (TU) determined on BHK-21 cells. Cells were processed for X-gal staining 3 days after treatment. At an MOI of 0.1 (white bars), 20.76 ± 1.1% of the cells were positive following transduction with the Rabies PV-pseudotyped vector, while only 11.56 ± 0.78% of the cells treated with the DuvSAF vector were positive. The LagNGA and the EBL1-pseudotyped vectors transduced 1.32 ± 0.20% and 1.25 ± 0.18% of cells, respectively. At an MOI of 1 (black bars), a similar pattern of transduction was observed. The Rabies PV and the DuvSAF vectors transduced 36.78 ± 1.05% and 13.13 ± 1.14% of the cells. The EBL1 pseudotype and the LagNGA vectors transduced 3.17 ± 0.35% and 2.79 ± 0.09% of the cells, repsectively. The transduction efficiency of Rabies PV-treated cells was the only one that increased as a result of a higher MOI (* p < 0.05).

To first determine the efficiency with which these vectors could transduce neuronal cells, we used SH-SY5Y cells. In this experiment, vector MOIs were adjusted based on functional titers (TU). Overall, Rabies PV-pseudotyped vectors performed better than the other pseudotypes in this assay, transducing 20.76 (± 1.1) and 36.78 (± 1.05) % of the cells at MOIs 0.1 and 1, respectively (Figure 1). Moreover, it was interesting to note that only the transduction efficiency of Rabies PV-treated cells significantly increased as a result of a higher MOI (two-way ANOVA, p < 0.05).

#### Transduction efficiencies of titer-adjusted lentiviral vector pseudotypes using particle titers

**Figure 2:**
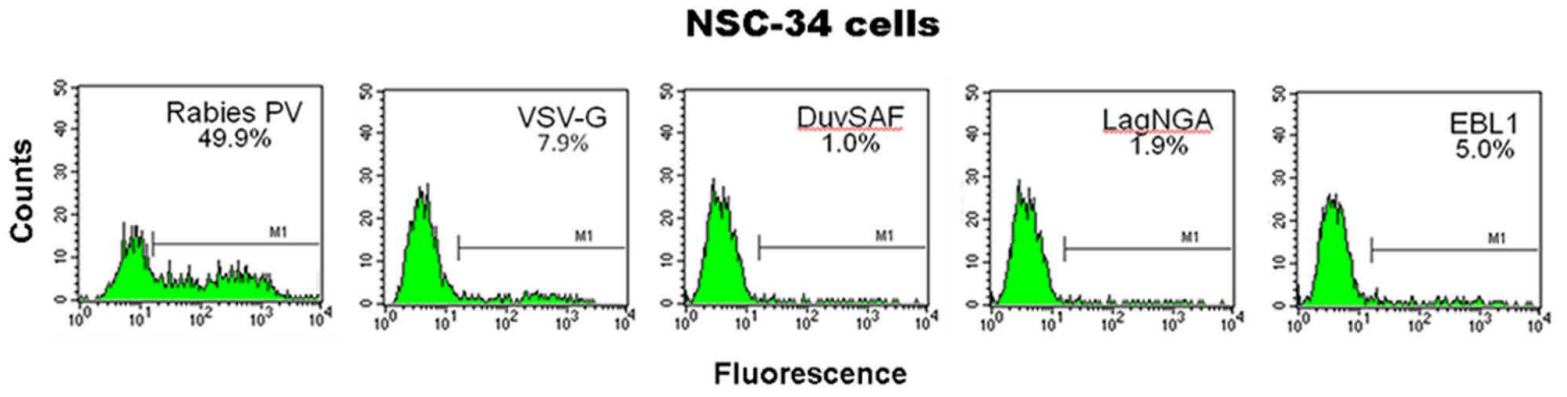
**Transduction of NSC-34 cells using lentiviral vectors pseudotyped with different lyssavirus glycoproteins**. NSC-34 neuroblastoma-spinal cord hybrid cells (2 × 10^5 ^cells) were transduced using EGFP-encoding lentiviral vectors pseudotyped with Rabies-PV, DuvSAF, LagNGA or EBL1 glycoproteins. A VSV-G vector was also used for comparison this time. MOIs were adjusted based on particle titers determined using virion RNA and 5 × 10^3 ^vector particles per cell were used. Cells were processed for FACS 3 days later. The Rabies PV pseudotypes transduced 49.9% of NSC-34 cells, while the performance of all other pseudotypes was considerably lower. The VSV-G vector transduced 7.9% of the cells, followed by the EBL1 (5.00%), LagNGA (1.9%) and DuvSAF (1.0%).

In this set of experiments, MOIs were adjusted based on particle titers determined by quantitative RT-PCR of virion-derived RNA to rule out effects caused by differences in receptor levels in target cells. Particle titer-adjusted pseudotypes were tested in the NSC-34 neuroblastoma-spinal cord hybrid cell line [24]. Vectors encoding EGFP were used to facilitate the analysis of transduced cells by flow cytometry (FACS). HOS cells that are easily transduced by lentiviral vectors [30,36] were tested in parallel to compare the performance of the various pseudotypes (data not shown). Cells were processed for FACS 3 days after treatment as described [33]. The results presented in Figure 2 indicate that at an MOI of 5 × 10^3 ^vector particles per cell, Rabies PV pseudotypes transduced 49.9% of NSC-34 cells, while the performance of all other pseudotypes was considerably lower, including a VSV-G pseudotype that was used for comparison.

#### Relative transduction efficiencies of lyssavirus-pseudotyped lentiviral vectors in E15 rat mixed spinal cord cultures

**Figure 3:**
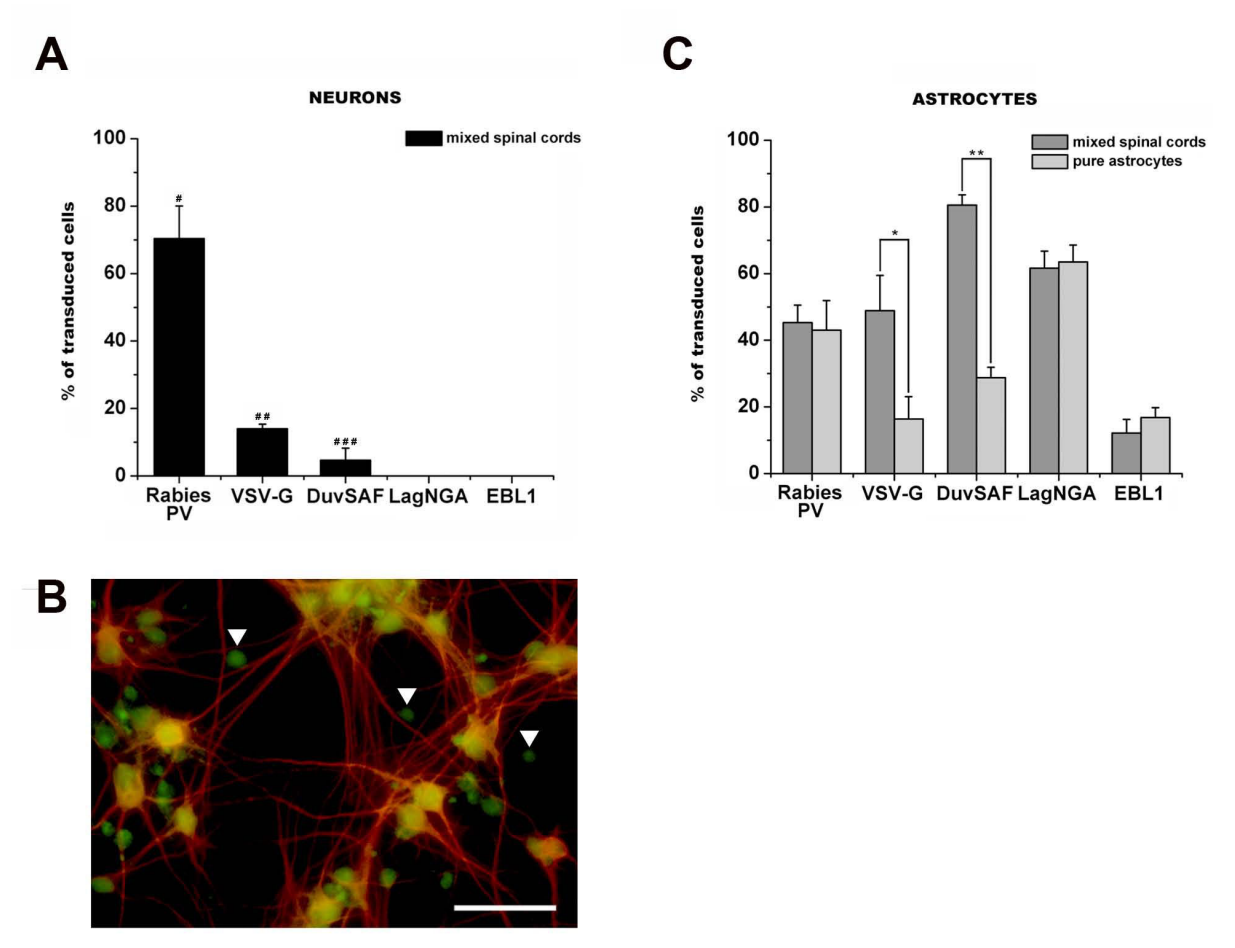
***In vitro *transduction of neurons and glia by lentiviral vectors pseudotyped with different lyssavirus glycoproteins**. Primary cell cultures were transduced using pseudotyped vectors encoding β-Gal (10^3 ^vector particles per cell) or EGFP (10^4 ^vector particles per cell) and processed 3 days later for cell identification. **A) **Quantification of the percentage of transduced neurons in primary embryonic mixed spinal cord cultures demonstrated that the Rabies PV pseudotype transduced 70.35 ± 9.66% of the neurons. The VSV-G vector transduced 13.98 ± 1.39% of the neurons and the DuvSAF-pseudotyped vector transduced 4.68 ± 3.58% of the cells. No transduced neurons could be detected in the LagNGA and EBL1 conditions. The difference between vectors reached significance in all groups (#, ##, ### p < 0.05). **B) **Immunocytochemistry data revealing the neuronal pattern of transduction observed after treatment with the Rabies PV pseudotype in primary mixed spinal cord cultures. Cells were stained for Map-2 (red) for identification of EGFP-positive neurons. Arrowheads indicate transduced astrocytes (GFP-positive/Map-2 negative cells). Scale bar = 50 μm. **C) **Quantification of the percentage of transduced astrocytes in mixed spinal cord (dark gray bars) *vs*. pure astrocyte cultures (light gray bars) demonstrated that all pseudotypes have the ability to transduce astrocytes with no effect of the culture type (mixed or pure) in the Rabies PV, LAgNGA, and EBL1 conditions, but a statistically significant decrease effect in the VSV-G and DuvSAF groups (*, ** p < 0.05).

The vectors were then tested in mixed spinal cord cultures to assess their potential specificity for neurons as opposed to glial cells. Cultures from 15 day-old rat embryos were transduced and processed 3 days later for cell identification. Two different kinds of vectors were used: vectors encoding β-Gal (10^3 ^vector particles per cell) and vectors encoding EGFP (10^4 ^vector particles per cell). Overall, the transduction efficiencies in mixed spinal cord cultures were similar to those observed in SH-SY5Y and NSC-34 cells, i.e. vectors pseudotyped with the Rabies PV glycoprotein revealed the highest transduction efficiency to neurons (70.35 ± 9.66% of transduced cells) (Figures 3A and 3B) followed by the others. The difference between vectors reached significance in all groups (p < 0.05).

Because all the vectors were able to transduce glial cells at some level in mixed cultures (Figure 3C – dark gray bars), we decided to investigate this effect in more detail using primary *pure *astrocytes. Indeed the results presented in Figure 3C show that all pseudotypes have the ability to transduce astrocytes in either mixed or pure astrocyte populations. Interestingly, the effect of the culture type (mixed or pure) did not affect the pattern of transduction in the Rabies PV, LAgNGA, and EBL1 conditions. The percentage of transduced astrocytes by VSV-G and DuvSAF pseudotyped vectors, however, significantly decreased in pure astrocytes compared to mixed cultures (Figure 3C). A two-way ANOVA analyzing vector and culture type revealed a significant effect in these particular groups (*, ** p < 0.05).

### Retrograde transport of vector particles

#### Uptake and retrograde transport *in vitro *using Campenot chambers

**Figure 4:**
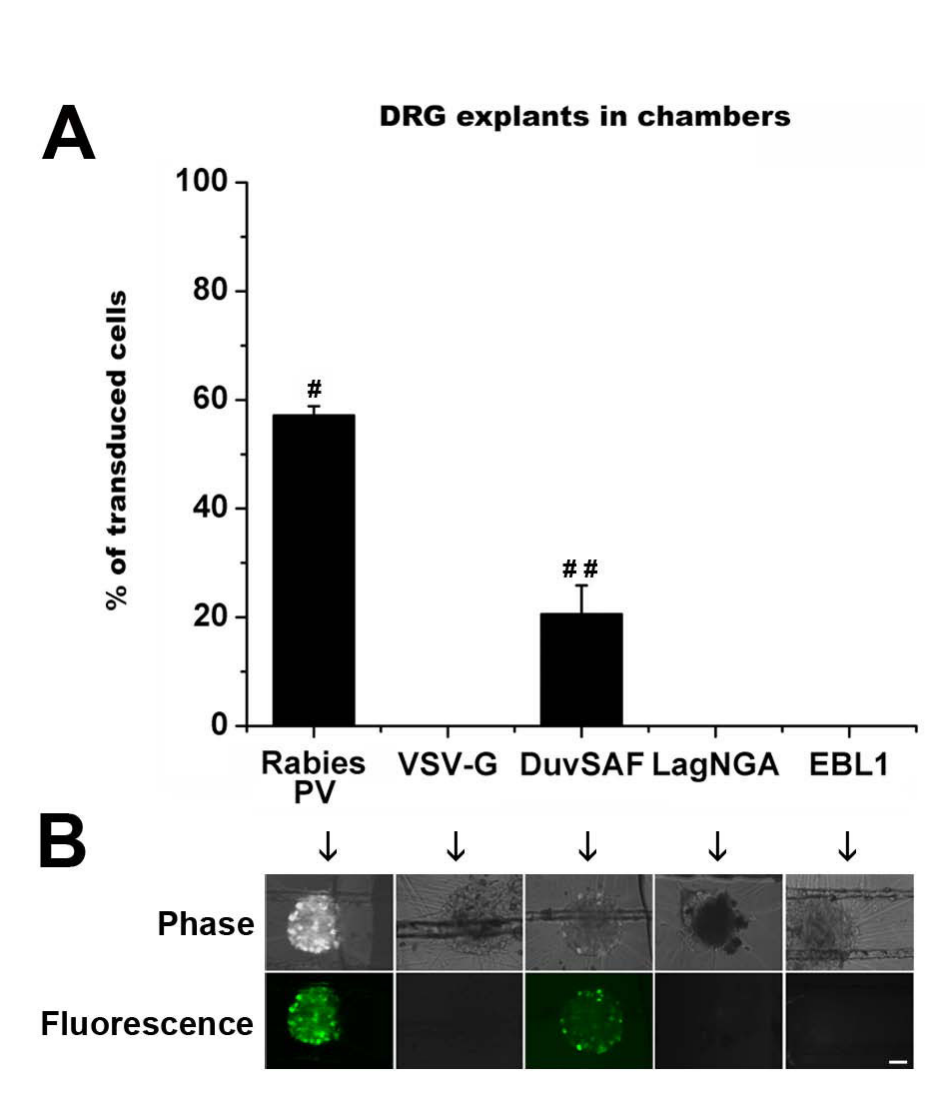
***In vitro *uptake and retrograde transport of pseudotyped lentiviral vectors**. DRG explants were plated in the central compartment of compartmentalized chambers. Left and right side compartments were filled with growth media supplemented with NGF to coax neurite growth into these compartments. The right compartments containing axon terminals were treated with equivalent concentrations of Rabies PV, DuvSAF, LagNGA, or EBL1-pseudotyped vectors (2 × 10^8 ^vector particles total) and fluorescence was analyzed 4 days later. A VSV-G pseudotyped vector was used as a negative control. For quantification of transduction, the fluorescence pixel intensity within the DRG cell bodies was quantified. Fluorescence could only be detected in DRG explants of chambers treated with the Rabies PV (57.12 ± 1.70%) and the DuvSAF (20.6 ± 5.27%) vectors, indicating their exclusive ability to be taken up and be retrogradely transported to the cell bodies of the DRG explants as opposed to the other pseudotypes. Differences between vectors were statistically significant in all groups (#, ## p < 0.05). Scale bar = 20 μm.

Aiming at using these pseudotypes as vehicles for CNS targeting by peripheral injection, we then evaluated their uptake when peripherally applied to axons terminals. For this purpose, DRG explants grown in compartmented chambers were used. The MOIs of vectors were normalized based on particle titers and 10 μl (2 × 10^8 ^vector particles total) of each pseudotyped vector were added to the chambers. Only one outer compartment of the chambers containing axon terminals was treated and, 3 days later, fluorescence was assessed in the central compartment containing the cell bodies. When vectors were directly added to the inner compartment containing the DRG cell bodies, fluorescence could be detected with all the pseudotypes (data not shown). On the other hand, when vectors were added to the compartments containing the axon terminals, fluorescence could only be detected in DRG explants of chambers treated with the Rabies PV and the DuvSAF vectors (Figure 4), indicating their ability to be taken up and be retrogradely transported to the cell bodies of the DRG explants.

#### Uptake and retrograde transport *in vivo*

**Figure 5:**
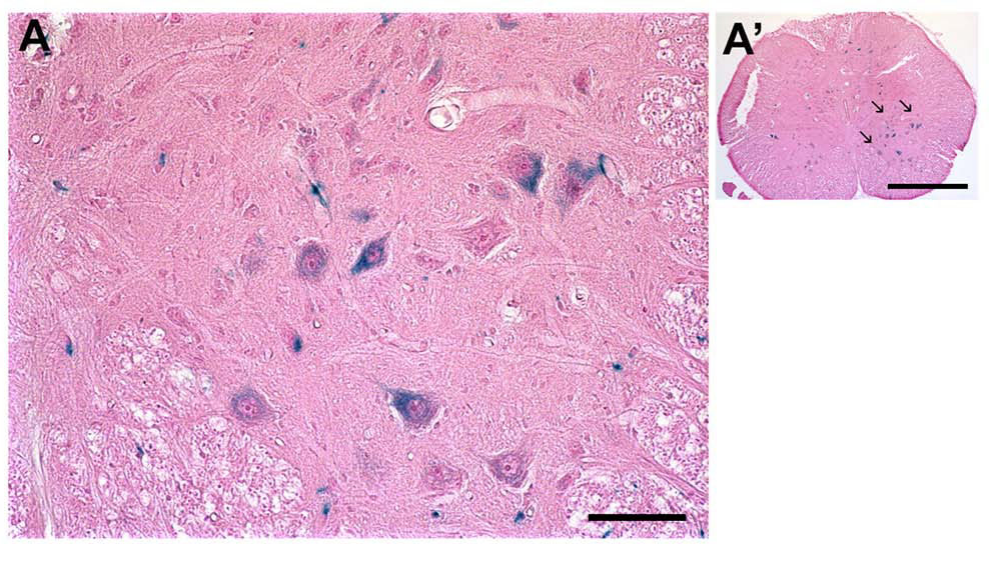
***In vivo *uptake and retrograde transport of the Rabies PV-pseudotyped lentiviral vector**. A total of 2.5 × 10^4 ^TU in a volume of 2 μl were injected into the crushed sciatic nerve of rats. Animals were euthanized after 4 weeks. An X-gal staining kit was used to detect cells expressing the transgene. Eosin was used for counter-staining. While all the vectors mediated some kind of retrograde transport to DRGs, β-Gal transgene expression could only be detected in motor neurons of the spinal cord (arrows) of animals injected with the Rabies PV-pseudotyped vector. Scale bars = 50 μm (A), 1 mm (A').

In order to assess the uptake and retrograde axonal transport of our pseudotyped lentiviral vectors *in vivo*, we next used the remote viral gene delivery model [37,41]. 2.5 × 10^4 ^TU of the different pseudotypes in a volume of 2 μl were injected into the crushed sciatic nerve of rats. To allow optimal transgene expression, animals were euthanized after 4 weeks and sciatic nerves, ipsilateral lumbar DRGs, and lumbar spinal cords were removed for analysis. Sections were stained using X-Gal to visualize β-Gal expressing cells. While all the vectors mediated some kind of retrograde transport to DRGs *in vivo*, β-Gal transgene expression was only observed in ventral horn motor neurons of the spinal cords injected with the Rabies PV-pseudotyped vector (Figure 5).

## Discussion

We have shown that HIV-1-based lentiviral vector particles can be pseudotyped with glycoproteins from different members of the *Lyssavirus *genus/*Rhabdoviridae *family. By analyzing the performance of these pseudotyped vectors using particle titer-adjusted vector stocks, we have demonstrated their distinct patterns of transduction *in vitro*. Finally, uptake and retrograde axonal transport have been evaluated both *in vitro *using compartmentalized chambers and *in vivo *using sciatic nerve injections. We were able to consistently reproduce our initial observations throughout the assays and, among all the pseudotypes tested, the Rabies PV virus-derived G glycoprotein [23] possessed the best performance and neuronal tropism. We believe that the assays described herein will be generally useful to characterize the retrograde transport characteristics of other pseudotypes in the future.

Retrograde transport of gene therapy vectors offers a potentially powerful strategy for targeting specific neuronal populations *in vivo*. A variety of pseudotyped lentiviral vectors have been demonstrated to transduce neuronal cells and to undergo retrograde transport [18,42]. In rodents, through direct or peripheral delivery, these studies have compared the CNS transduction patterns of different EIAV- and HIV-1-based vector pseudotypes. Overall, vectors pseudotyped with VSV-G or Rabies virus G-glycoproteins including those from the Evelyn-Rokitnicki-Abelseth (ERA) strain [8,15] or the challenge virus standard strains CVS, CVS-B2c and CVS-N2c [8,17,43] were shown to preferentially transduce neurons. However, transduction of astrocytes with ERA-pseudotyped EIAV vectors was also apparent [8,15]. The main difference reported was the ability of Rabies-G but not VSV-G pseudotyped vectors to undergo retrograde transport to appropriate distal neurons of the lumbar spinal cord after peripheral delivery [8,15,17]. In summary, these results demonstrated that targeted transduction in the CNS can be achieved using specific glycoproteins to pseudotype lentiviral vectors. Moreover, EAIV vectors pseudotyped with the ERA and CVS glycoproteins [8,15] and HIV-1 vectors pseudotyped with the CVS-B2c glycoprotein [17] have been proven to be attractive candidates when MNs are the main target. One problem with the above studies is that different assays were used to determine vector titers, making a direct comparison of the results difficult. Some of the assays used involved functional (biological) titers determined on a variety of different cell lines including canine osteosarcoma (D17) cells [8] and human 293T cells [43]. Other assays involved p24 antigen levels determined by ELISA [17,20]. While functional titers determined on heterologous cell lines may underestimate vector titers, particle assays based on p24 can be misleading because they do not necessarily reflect intact vector particles only [27].

Based on the transduction experiments conducted with SH-SY5Y, NSC-34 and mixed spinal cord and astrocyte cultures, there appear to be differences among the various pseudotypes tested in terms of cellular tropism, possibly due to differences in the receptors used by the various pseudotypes. A variety of putative cellular receptors have been described for Rabies virus including the acetylcholine receptor and the low-affinity nerve-growth factor receptor (P75NTR) [44]. However, there appear be additional receptors as lentivector pseudotypes bearing Lyssavirus glycoproteins have been shown to be capable of transducing a variety of cell lines and primary cells supposedly lacking the above receptors [8,17,36]. The use of cell specific-promoters can restrict distribution and specific gene expression to the desired populations.

EIAV-based lentiviral vectors have been recently used to peripherally deliver therapeutic proteins on mouse models of familial ALS and SMA [45,46]. Although successful in young mice, the efficiency of these approaches remains to be determined in larger species. It is currently unknown whether in the context of gene therapy trials in humans these vectors will ultimately be able to transduce a sufficient number of MNs to make a therapeutic impact. Thus, further attempts aimed at improving the retrograde transport of these vectors will be necessary.

Lentiviral vectors have reached the clinical trial stage [47] and clinical translation of such vectors aimed at treating MN disorders may ultimately be feasible. Lentiviral vectors combine the advantages of long-term transgene expression, minimal immunogenicity, ability to accommodate larger transgenes, and the capacity to form pseudotypes with a wide variety of different glycoproteins. Thus, this novel Rabies PV G-pseudotyped HIV-1-based vector might be an important vehicle for remote gene delivery in the treatment of MN diseases, pain, and neuropathy.

## Conclusion

Our results indicate that HIV-1-based lentiviral vectors pseudotyped with the Rabies PV glycoprotein might provide important vehicles for CNS targeting by peripheral injection in the treatment of motor neuron diseases, pain, and neuropathy.

## Competing interests

The authors declare that they have no competing interests.

## Authors' contributions

NB and JR conceived and designed the experiments. RK helped with lentiviral vector production. NT and XYZ worked on the construction of Lyssavirus glycoprotein-encoding plasmids. TF and HK performed the *in vitro *and *in vivo *transduction experiments. NB, TF and JR wrote the manuscript. All authors read and approved the final manuscript.
